# *N-*terminal BET bromodomain inhibitors disrupt a BRD4-p65 interaction and reduce inducible nitric oxide synthase transcription in pancreatic β-cells

**DOI:** 10.3389/fendo.2022.923925

**Published:** 2022-09-13

**Authors:** Joshua A. Nord, Sarah L. Wynia-Smith, Alyssa L. Gehant, Rachel A. Jones Lipinski, Aaron Naatz, Inmaculada Rioja, Rab K. Prinjha, John A. Corbett, Brian C. Smith

**Affiliations:** ^1^ Department of Biochemistry, Medical College of Wisconsin, Milwaukee, WI, United States; ^2^ Immuno-Epigenetics, Immunology Research Unit, GlaxoSmithKline Medicines Research Centre, Stevenage, United Kingdom

**Keywords:** BET bromodomain, BRD4, diabetes, small molecule inhibitor, iNOS, nitric oxide, NF-κB, bioluminescence resonance energy transfer

## Abstract

Chronic inflammation of pancreatic islets is a key driver of β-cell damage that can lead to autoreactivity and the eventual onset of autoimmune diabetes (T1D). In the islet, elevated levels of proinflammatory cytokines induce the transcription of the inducible nitric oxide synthase (iNOS) gene, *NOS2*, ultimately resulting in increased nitric oxide (NO). Excessive or prolonged exposure to NO causes β-cell dysfunction and failure associated with defects in mitochondrial respiration. Recent studies showed that inhibition of the bromodomain and extraterminal domain (BET) family of proteins, a druggable class of epigenetic reader proteins, prevents the onset and progression of T1D in the non-obese diabetic mouse model. We hypothesized that BET proteins co-activate transcription of cytokine-induced inflammatory gene targets in β-cells and that selective, chemotherapeutic inhibition of BET bromodomains could reduce such transcription. Here, we investigated the ability of BET bromodomain small molecule inhibitors to reduce the β-cell response to the proinflammatory cytokine interleukin 1 beta (IL-1β). BET bromodomain inhibition attenuated IL-1β-induced transcription of the inflammatory mediator *NOS2* and consequent iNOS protein and NO production. Reduced *NOS2* transcription is consistent with inhibition of NF-κB facilitated by disrupting the interaction of a single BET family member, BRD4, with the NF-κB subunit, p65. Using recently reported selective inhibitors of the first and second BET bromodomains, inhibition of only the first bromodomain was necessary to reduce the interaction of BRD4 with p65 in β-cells. Moreover, inhibition of the first bromodomain was sufficient to mitigate IL-1β-driven decreases in mitochondrial oxygen consumption rates and β-cell viability. By identifying a role for the interaction between BRD4 and p65 in controlling the response of β-cells to proinflammatory cytokines, we provide mechanistic information on how BET bromodomain inhibition can decrease inflammation. These studies also support the potential therapeutic application of more selective BET bromodomain inhibitors in attenuating β-cell inflammation.

## Introduction

Type 1 and type 2 diabetes (T1D and T2D, respectively) are driven, at least partly, by inflammation (1, 2). The proinflammatory cytokine interleukin 1 beta (IL-1β) is a key initiator in this inflammatory process as elevated IL-1β levels in pancreatic islets are associated with the onset and progression of T1D and T2D (3–5). Pancreatic islets harbor a heterogeneous population of endocrine hormone-producing cells such as α-, δ-, and β-cells and non-endocrine immune cells such as macrophages. In response to adverse stimuli, IL-1β is produced and secreted by activated macrophages (6, 7). Elevated concentrations of IL-1β signal *via* IL-1 receptors on β-cells (8) to increase NF-κB-driven transcription of inflammatory genes, including *NOS2* (9–11), a process that can be further potentiated by interferon gamma (IFN-γ) (12). β-cells express *NOS2* (9–11) and produce nitric oxide (NO) at levels similar to activated macrophages (13–17). In β-cells, NO inhibits mitochondrial oxidative phosphorylation and aconitase, resulting in inhibition of glucose-stimulated insulin secretion (GSIS) (18–22), antiproliferative response (23), and β-cell DNA damage (17). Prolonged exposure to NO can result in β-cell death (18). Despite the damaging effects, NO can also promote β-cell survival (17, 24). These disparate consequences of NO in β-cells likely depend upon the temporal NO concentration, with higher concentrations and longer durations being pathological. Indeed, constitutive levels of NO production are beneficial and required for physiological processes in β-cells (25). In contrast, high NO concentrations can cause excessive, chronic, damaging inflammation and may contribute to autoimmune diabetes pathology (26).

The mechanisms responsible for inflammation-driven diseases are poorly understood, but they are generally accompanied by changes in epigenetic control, chromatin landscape, and altered transcriptional regulation (27). Because of these changes, modulation of epigenetic factors and transcriptional regulators that decrease the transcription of inflammatory mediators is a growing area of therapeutic development. In this regard, the bromodomain and extraterminal domain (BET) family of epigenetic regulatory proteins (BRD2, BRD3, BRD4, and BRDT) have emerged as promising drug targets in a wide variety of inflammatory contexts [reviewed in (28)]. The BET family of proteins is characterized by two *N*-terminal bromodomains (BD1 and BD2) and a *C*-terminal extraterminal domain. Bromodomains bind acetyl-lysine residues (29, 30), allowing interaction with acetylated histone tails and acetylated non-histone proteins such as transcription factors. BRD2, BRD3, and BRD4 are ubiquitously expressed, including in pancreatic β-cells (31), and therefore of particular interest herein. Inhibition of BET bromodomain proteins affords broad anti-inflammatory effects in diseases such as arthritis (32), fibrosis (33, 34), coronary artery disease (35), cardiovascular disease (36), and diabetes (37, 38). However, clinical trials using small molecule inhibitors of BET bromodomains in the context of cancer have yet to be successful [reviewed in (39)]. Dose-limiting toxicities and adverse side effects have hindered the advancement of several BET bromodomain inhibitors in clinical studies [reviewed in (40, 41)]. These issues are likely the result of poor selectivity, as most BET inhibitors being used in clinical trials inhibit both bromodomains (BD1 and BD2) of all four BET bromodomain proteins (pan-BET bromodomain inhibitors).

In T1D, pan-BET bromodomain inhibitors prevent the onset and progression of insulitis, a hallmark of T1D, in the non-obese diabetic (NOD) mouse model (37, 38, 42). Although the protective mechanisms of pan-BET bromodomain inhibitors are poorly understood, inhibiting NF-κB signaling (37) may be central to these protective mechanisms. In several disease models, including cancer, inhibition of BET bromodomains attenuates NF-κB-mediated transcription (43, 44). BET bromodomains bind the acetylated NF-κB subunit p65 and modulate promoter activity by increasing the transcriptional transactivation activity of p65 [reviewed in (45)]. BET proteins, particularly BRD4, can recruit and interact with initiation and elongation complexes, such as Mediator and positive transcription elongation factor b (P-TEFb), at promoter and enhancer regions to activate transcription [reviewed in (45, 46)]. This mechanism highlights the importance of p65 acetylation, which has been identified as a principal component in modulating NF-κB-driven transcriptional outputs (47, 48). Many of the reported effects of BET bromodomain inhibitors are not consistently observed across cell- and disease-specific contexts, highlighting the need to assess BET bromodomain inhibitor-driven outcomes in β-cells.

Using insulinoma INS 832/13 cells and isolated rat pancreatic islets, we examined the role of BET proteins in regulating proinflammatory gene expression following IL-1β stimulation. We show that BET bromodomain inhibitors attenuate IL-1β-induced *NOS2* expression and NO production. This effect was associated with a decrease in NF-κB transcriptional activity through inhibition of BET bromodomains that did not disrupt the NF-κB signaling cascade in the cytoplasm. Using bioluminescent resonance energy transfer (BRET) binding analyses, we identified that BET bromodomain inhibitors disrupt the interaction of the BRD4 tandem bromodomains and p65 and that selective inhibition of the first bromodomain of BRD4 was sufficient to abolish this interaction. Finally, we show that IL-1β-mediated attenuation of mitochondrial oxygen consumption rates and β-cell viability are partially rescued by pan-BET or BD1-selective inhibitors.

## Materials and methods

### Cell culture

INS 832/13 were cultured in RPMI 1640 supplemented with 10% v/v fetal bovine serum (FBS, Gemini Bio), 2 mM L-glutamine, 1 mM sodium pyruvate, 10 mM HEPES, 50 µM 2-mercaptoethanol, 100 units/mL penicillin, and 100 μg streptomycin. Cells were grown at 37°C and 5% CO_2_. At confluency (~70-80%), cells were lifted using 0.05% w/v trypsin and plated at 500,000 cells/mL for experimental treatments unless otherwise noted.

### Isolation and culture of primary rat islets

Pancreatic islets were harvested from male Sprague Dawley rats (Charles River) *via* collagenase digestion as previously described (49). Isolated islets were cultured in CMRL-1066 (ThermoFisher, Waltham, MA) supplemented with 10% v/v FBS, 2 mM L-glutamine, 100 units/mL penicillin, and 100 μg streptomycin. For RT-qPCR experiments, 60-80 islets were used per treatment condition in 300 μL CMRL-1066. For immunoblot experiments, ~100 islets were used per treatment condition. All studies were conducted in accordance with the GlaxoSmithKline (GSK) Policy on the Care, Welfare, and Treatment of Laboratory Animals and were reviewed by the Institutional Animal Care and Use Committee either at GSK or by the ethical review process at the Medical College of Wisconsin, where the work was performed.

### RNA isolation and real-time quantitative polymerase chain reaction analysis

Total RNA was isolated from INS 832/13 cells and primary rat islets after indicated treatment conditions using the RNeasy mini kit (Qiagen, Germantown, MD). Isolated RNA samples were treated with DNase using a TURBO DNA-free kit (Invitrogen, Carlsbad, CA). Complementary DNA (cDNA) was synthesized using Maxima H Minus Reverse Transcriptase (Thermo Scientific, Waltham, MA). Using these cDNA as templates, RT-qPCR analysis was completed using primers listed in **Supplementary Table 1**, obtained from Integrated DNA Technologies (IDT, Coralville, IA), EvaGreen qPCR master mix (Midwest Scientific, Valley Park, MO), and run on a CFX96 Real-Time System (Bio-Rad, Hercules, CA) with the following cycle conditions: 95°C for 3 minutes, then 40 cycles of 95°C for 15 seconds, 64°C for 30 seconds, and 72°C for 35 seconds. Each gene was assayed in 3 to 5 independent biological replicates with GAPDH used for normalization. Data is normalized to an internal reference condition for fold change (2^-ΔΔCT^).

### Immunoblotting

Cells or islets were lysed in RIPA buffer containing 150 mM NaCl, 50 mM Tris, 1% v/v IGEPAL, 0.1% v/v SDS, and phenylmethylsulfonyl fluoride (PMSF) and subjected to sonication *via* a Bioruptor Plus (Diagenode, Denville, NJ). Extracts were run on Mini-PROTEAN TGX Stain-Free Protein Gels (Bio-Rad) before being transferred to nitrocellulose membrane using a Trans-Blot Turbo fast transfer system (Bio-Rad). The membranes were blocked in 2.5% w/v BSA or 2.5% w/v milk in PBST before incubation with a primary antibody. Membranes were washed with PBST before incubation with HRP-conjugated or fluorescently-labeled secondary antibodies. The membranes were rewashed before imaging. Bands were detected with chemiluminescence (50) and visualized on ChemiDoc MP Imaging System (Bio-Rad). Antibodies used for Immunoblot detection are listed in **Supplementary Table 2**.

### Subcellular fractionation

Fractionation of INS 832/13 cells was completed using a modified REAP method (51). Briefly, cells were washed with ice-cold PBS, scraped, and subjected to a ‘pop-spin’ (10 seconds in a table-top Eppendorf centrifuge) to pellet. The cell pellet was resuspended in 450 μL ice-cold NP-40 (0.1% v/v). 150 μL of cell suspension was saved as the whole-cell fraction. An additional pop-spin was completed, and 150 μL supernatant was saved as the cytoplasmic fraction. The remaining supernatant was removed, and the remaining cell pellet containing nuclei was resuspended in 100 μL of ice-cold NP-40 (0.1% v/v) and saved as the nuclear fraction. Samples were flash-frozen in liquid nitrogen and stored at -80°C until further processing. Whole-cell and nuclear fractions were subjected to sonication *via* a Bioruptor Plus (Diagenode) before an additional spin (>10,000 × *g* for 3 minutes at 4°C) to remove cell debris. Samples were analyzed by immunoblot as described above.

### Chemical inhibition of BET bromodomains and IL-1β treatments

BET bromodomain inhibitors were dissolved in DMSO; suppliers are listed in **Supplementary Table 3**. An equal volume of DMSO was used as a control. All treatments were completed in ≤0.5% v/v final DMSO concentration in the media. A concentration of 0.5-1 µM (+)-JQ1 was used for cell culture treatments based on previously published concentration-response experiments (52, 53). A concentration of 1-2 µM of I-BET151, GSK778, GSK789, or GSK046 was used for cell culture treatments as recommended by our collaborators at GlaxoSmithKline (54–56). Recombinant IL-1β (Peprotech, Cranbury, NJ) was reconstituted RPMI at 0.1 μg/mL, which we determined was the equivalent of 1000 units/mL (U/mL) *via* in-house Griess assay. All IL-1β treatments were conducted at 5 U/mL.

### Nitrite detection

Nitric oxide generation was assessed by measuring nitrite accumulation in the culture medium as previously described (57). Briefly, equal amounts of the Griess reagents and culture medium were combined, and the absorbance was read at 540 nm. Nitrite concentrations were calculated from a nitrite standard curve, and data are presented as pmol/2000 cells plated.

### Immunostaining

Cells were fixed using freshly dissolved 4% v/v paraformaldehyde in PBS. Cells were permeabilized with 0.4% v/v Triton X-100 and 0.1% w/v sodium citrate before blocking with 5% w/v BSA in PBS for 25 minutes at room temperature. Primary antibodies were applied in a 1% w/v BSA in PBS overnight at 4°C. Coverslips were washed 3× with PBS, secondary antibodies were applied for 2 hours at room temperature, and DAPI was used as a counterstain. Immunofluorescence micrographs were captured using Nikon Ti2 CSU W1 Imaging System (Nikon Instruments, Melville, NY). Images collected from experimentally treated samples and controls were identically processed. All primary and secondary antibodies are listed in **Supplementary Table 2**.

### Luciferase reporter assays

INS 832/13 cells were transfected with 0.35 μg of p1242 3x-KB-L plasmid (a gift from Bill Sugden; Addgene plasmid #26699; http://n2t.net/addgene:26699; RRID : Addgene_26699) (58) per 500,000 cells using Avalanche^®^-Omni transfection reagent (EZ-Biosystems, College Park, MD). Forty-eight hours after transfection, BET bromodomain inhibitors were added for 1 hour before adding IL-1β (5 U/mL) for 5 hours. Luciferase activity was determined *via* the Luciferase Assay System (Promega Corporation, Madison, WI) and read on BioTek Cytation 5 multi-mode microplate reader using a Luminescence filter cube (BioTek, Winooski, VT). DMSO vehicle (≤0.5% v/v) with 5 U/mL IL-1β was used as an internal control and signal set equal to 100%.

### Generation of Sirt1 knockout INS 832/13 cells by CRISPR-Cas9

Two sgRNAs specific for rat *Sirt1* were separately cloned into the pX462 Cas9n double nickase vector according to Zhang et al. (59). sgRNA sequences used to target *Sirt1* were 5′-GTCATCGTCATCACTTTCAC-3′ and 5′-CACACGCAAGCTCTAGTGAC-3′. INS 832/13 cells were simultaneously transfected with both plasmids using Lipofectamine 2000 Transfection Reagent (ThermoFisher Scientific, Waltham, MA), and transformants were selected with 1 μg/mL puromycin. After 10-14 days, individual colonies were picked and transferred to larger cultures. Clones were screened for Sirt1 protein by immunoblot analysis. Potential knockout clones were genome sequenced (Functional Biosciences, Madison, WI). The *Sirt1^-/-^* INS 832/13 cells had early truncations in both gene copies (D166/D167).

### Nanoluciferase bioluminescence resonance energy transfer (NanoBRET)

INS 832/13 cells were plated in a 6-well plate at 500,000 cells per well and incubated for 18 hours at 37°C. Media was replaced with OptiMEM reduced serum medium (ThermoFisher). Cells were transfected with 4 μg of HaloTag-BRD3 tandem bromodomain construct (residues 24-416), HaloTag-BRD4 tandem bromodomain construct (residues 38-460), or a HaloTag construct without bromodomains in the pFN21A plasmid (gifts from Danette Daniels, Promega) and 0.4 μg of full-length NanoLuc-p65 in the pFN31K plasmid (custom cloning by Genscript) using Avalanche^®^-Omni transfection reagent (EZ-Biosystems). After 24 hours, transfected cells were replated onto cell culture-treated white 96-well plates (Corning #3903) at 900,000 cells per well in complete culture medium. Cells were allowed to adhere for 1 hour at room temperature, and then plates were transferred to a 37°C incubator for 4 hours. BET bromodomain inhibitors or DMSO vehicle were added from 20× stocks made fresh in complete medium. HaloTag-specific ligand Janelia Fluor 585 (Janelia Materials #JF585) (60) or vehicle was added to a final 100 nM concentration from 20× stock made fresh in complete medium. Following an 18-hour incubation at 37°C, media was replaced with OptiMEM containing no phenol red (ThermoFisher #11058021), and NanoBRET Nano-Glo Substrate (Promega #N1571) was added to all samples to a final concentration of 10 μM. Plates were read within 5 minutes using a BioTek Cytation 5 plate reader equipped with a custom NanoBRET filter cube (BioTek). The NanoBRET ratio was calculated using the emission ratio at 610 nm to 450 nm for three control wells subtracted from the 610/450 nm ratio for three JF585-containing wells. The averages of up to 12 NanoBRET ratio data points, multiplied by 1000, yielded the output signal expressed as milliBRET units. The error was calculated as SEM.

### Oxygen consumption rate

Oxygen consumption rates (OCR) were read on an XFe96 analyzer (Agilent, Santa Clara, CA). INS 832/13 cells were plated at a density of 4 × 10^4^ cells per well of an XF96 cell culture microplate (Agilent #101085-004) and allowed to adhere for 1 hour at room temperature. DMSO or BET bromodomain inhibitors were added for 2 hours before the addition of IL-1β (5 U/mL) for 24 hours. The oxygen consumption rate was measured. Hoechst 33342 solution (Thermo Scientific #62249) was added to a final concentration of 2 μM to stain nuclei. Plates were transferred to a BioTek Cytation 5 instrument equipped with a microscope and DAPI filter (BioTek). Stained cell nuclei were imaged with a 4× objective and counted with the built-in Cell Analysis module in the Gen5 software package. Normalized OCR is represented as pmol/minute per 1000 cells. Data represent the mean and standard deviation of a single time point from 7-8 wells per treatment group.

### Cell viability assay

Cell viability was measured using the neutral red assay described previously (61). Briefly, INS 832/13 cells were plated onto 24-well plates in 400 µL medium. After 1 hour of pretreatment with indicated BET bromodomain inhibitor, cytokines were added for 24 hours. Upon completion of cell treatments, neutral red was added at 40 µg/mL and incubated for 1 hour at 37°C to allow for cell uptake. The medium was removed before fixing cells with 200 µL formaldehyde (1% v/v in 1% w/v CaCl_2_). Neutral red was extracted with 200 µL of 50% ethanol containing 1% v/v acetic acid. Absorbance was read at 540 nm using a BioTek Cytation 5 multi-mode microplate reader.

## Results

### Pan-BET bromodomain inhibitors abrogate IL-1β induced expression of iNOS through inhibition of NF-κB transcriptional activity

**Figure 1:**
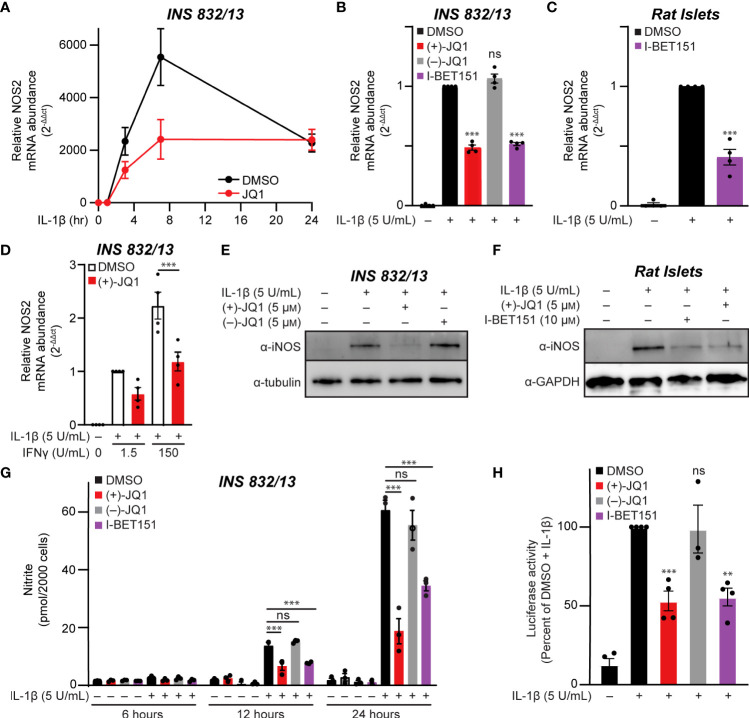
Pan-BET bromodomain inhibition mitigates IL-1β-induced transcription of *NOS2*, iNOS protein production, and nitrite accumulation in pancreatic β-cells. **(A)** RT-qPCR analysis of *NOS2* transcript levels in INS 832/13 cells treated for 1 hour with 0.5 μM (+)-JQ1 followed by 5 U/mL IL-1β exposure for 1, 3, 7, or 24 hours. **(B)** RT-qPCR analysis of *NOS2* transcript levels in INS 832/13 cells treated for 1 hour with 0.5 μM (+)-JQ1, 0.5 μM (–)-JQ1, 1 μM I-BET151, or DMSO followed by addition of 5 U/mL IL-1β for 3 hours. **(C)** RT-qPCR analysis of *NOS2* transcript levels in primary rat islets treated for 3 hours with 10 μM I-BET151 or DMSO followed by 5 U/mL IL-1β for 3 hours. **(D)** RT-qPCR analysis of *NOS2* transcript levels in INS 832/13 cells treated for 1 hour with 0.5 μM (+)-JQ1 or DMSO followed by 5 U/mL IL-1β and indicated concentration of IFN-γ for 3 hours. **(E)** Immunoblot with an antibody directed against iNOS protein or α-tubulin loading control in INS 832/13 cells treated for 1 hour with 0.5 μM (+)-JQ1, 0.5 μM (–)-JQ1, or DMSO followed by addition of 5 U/mL IL-1β for 20 hours. **(F)** Immunoblot using an antibody directed towards iNOS protein or β-actin loading control in primary rat islets treated for 3 hours with 5 μM (+)-JQ1, 10 μM I-BET151, or DMSO followed by the addition of 5 U/mL of IL-1β for 24 hours. **(G)** Griess assay for nitrite accumulation in INS 832/13 cells cultured for 6, 12, and 24 hours in the presence of IL-1β and 0.5 μM (+)-JQ1, 0.5 μM (–)-JQ1, 1 μM I-BET151, or DMSO. **(H)** NF-κB reporter assay in INS 832/13 cells transfected with a 3x-κB-Luciferase plasmid (Addgene #26699) treated with 0.5 μM (+)-JQ1, 0.5 μM (–)-JQ1, 1 μM I-BET151, or DMSO for one hour followed by addition of 5 U/mL IL-1β for 5 hours. All error bars for RT-qPCR, Griess assays, and luciferase reporter assays represent the standard error of the mean (SEM) from 3-5 independent biological replicates, except for panel A which contains 2-3 replicates per treatment group/time-point. Significance was determined by ANOVA followed by Dunnett’s multiple comparisons test, *< 0.05, **< 0.01, ***< 0.005 and ns > 0.05.

The temporal effects of pan-BET bromodomain inhibition on IL-1β-induced transcription of *NOS2* were examined using INS 832/13 cells. Cells were pre-treated with (+)-JQ1, a pan-BET bromodomain inhibitor (62), IL-1β was added for up to 24 additional hours, and *NOS2* mRNA accumulation was examined by RT-qPCR. In response to IL-1β, *NOS2* mRNA accumulation was first observed following a 3-hour IL-1β treatment and maximal following a 7-hour treatment (**Figure 1A**). IL-1β-induced *NOS2* expression was attenuated in the presence of (+)-JQ1 (**Figure 1A**). Like (+)-JQ1, a second structurally divergent pan-BET bromodomain inhibitor I-BET151 (54) attenuated IL-1β-induced *NOS2* mRNA accumulation while the inactive enantiomer of JQ1 [(–)-JQ1]) did not (**Figure 1B**). Using multiple structurally diverse pan-BET bromodomain inhibitors provides pharmacological evidence that targeted BET bromodomain inhibition attenuates IL-1β-induced *NOS2* expression in INS 832/13 cells. Like insulinoma cells, IL-1β stimulated *NOS2* expression in rat islets was attenuated in the presence of I-BET151 (**Figure 1C**). As IFN-γ amplifies IL-1β-mediated iNOS expression in β-cells (12), the effects of BET bromodomain inhibition were assessed in INS 832/13 cells exposed to IL-1β and IFN-γ. pan-BET inhibitor (+)-JQ1 attenuated *NOS2* transcription in the presence of IL-1β and IFN-γ (**Figure 1D**). Consistent with the decrease in mRNA, pan-BET bromodomain inhibitors decreased iNOS protein expression (as determined by immunoblot analysis) following IL-1β treatment in INS 832/12 cells (**Figure 1E**) and rat islets (**Figure 1F**) and NO accumulation (using nitrite as a proxy) at 12 and 24 hours following IL-1β treatment (**Figure 1G**). Since the activation of *NOS2* expression in response to proinflammatory signals such as IL-1β and tumor necrosis factor-alpha (TNFα) requires NF-κB signaling (9–11), the effects of BET bromodomain inhibition on NF-κB promoter activity were examined using a luciferase reporter plasmid containing three κB binding elements upstream of luciferase (3x-κB). Consistent with previous studies showing that BET bromodomains facilitate transcription of p65 target genes in response to TNFα (43, 44), (+)-JQ1 and I-BET151 attenuated NF-κB transcriptional activity in response to IL-1β by nearly 50% compared to DMSO and (–)-JQ1 controls (**Figure 1H**). These findings indicate that BET bromodomains are required for the full NF-κB activated transcription of *NOS2*, as shown by the observed BET bromodomain inhibitor-mediated reduction of *NOS2* transcript levels and NF-κB reporter activity (**Figures 1A–D, H**).

### BET bromodomain inhibitors attenuate NF-κB without disrupting IκBα degradation and transcription or abolishing p65 nuclear translocation in β-cells

**Figure 2:**
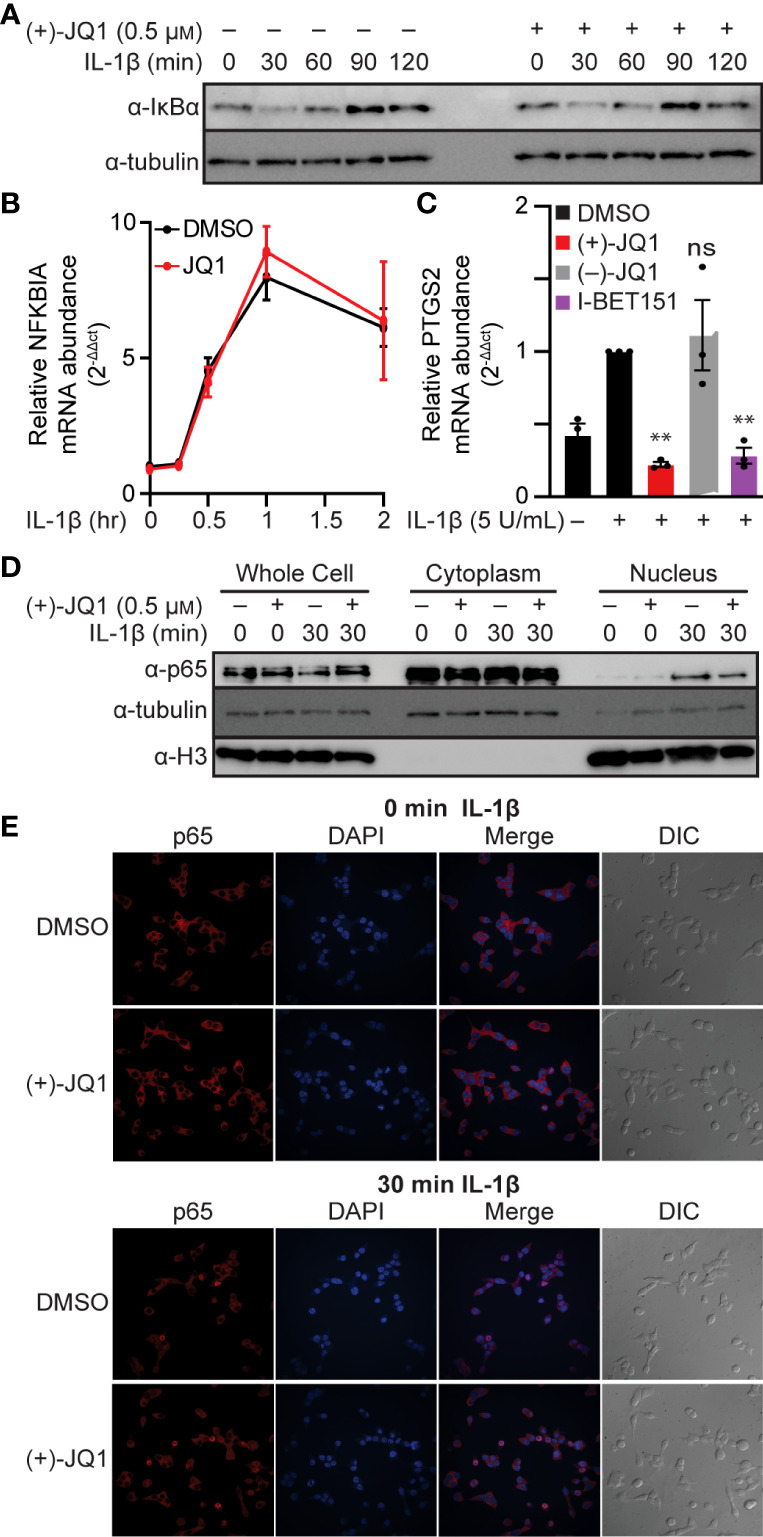
BET bromodomain inhibitors act by inhibiting transcriptional activation of select NF-κB gene targets. **(A)** Immunoblot using an antibody directed towards the IκBα protein in INS 832/13 cells treated for 1 hour with 0.5 μM (+)-JQ1 or DMSO followed by 5 U/mL IL-1β for up to 120 minutes. **(B)** RT-qPCR analysis of *NFKBIA* in INS 832/13 cells treated for 1 hour with 0.5 μM (+)-JQ1 or DMSO followed by 5 U/mL IL-1β for up to 2 hours. **(C)** RT-qPCR analysis of *PTGS2* in INS832/13 cells treated for 1 hour with 0.5 μM (+)-JQ1, 0.5 μM (–)-JQ1, 1 μM I-BET151, or DMSO followed by addition of 5 U/mL IL-1β for 3 hours. **(D)** Immunoblot analysis in INS 832/13 subcellular fractions using antibodies directed against p65 or α-tubulin or histone H3 loading controls after 1-hour pre-treatment with 0.5 μM (+)-JQ1 or DMSO followed by the indicated 0- or 30-minute exposure to 5 U/mL IL-1β. **(E)** Immunocytochemistry analysis of p65 in INS 832/13 cells upon 1-hour pre-treatment with 0.5 μM (+)-JQ1 or DMSO followed by 0- or 30-minute exposure to 5 U/mL IL-1β. Immunoblots are representative of two independent biological replicates. All error bars for RT-qPCR represent the standard error of the mean (SEM) from 3 independent biological replicates. Significance was determined by ANOVA followed by Dunnett’s multiple comparisons test, *< 0.05, **< 0.01, ***< 0.005, and ns > 0.05.

NF-κB is typically found in the cytoplasm in a complex with the inhibitory protein IκBα. Signaling pathways that stimulate IκBα phosphorylation and degradation allow NF-κB to translocate to the nucleus and bind chromatin [reviewed in (63, 64)]. As shown in **Figure 2A**, IL-1β stimulated the time-dependent loss of IκBα in INS 832/13 cells, first apparent following a 30-minute exposure. As a target of NF-κB transcriptional activation (64), IκBα returned to control levels following a 90-minute incubation with IL-1β. The pan-BET bromodomain inhibitor (+)-JQ1 did not alter this process (**Figure 2A**). Interestingly, (+)-JQ1 did not change transcriptional activation of the IκBα gene, *NFKBIA*, which peaked after one hour of IL-1β treatment, thus indicating that BET bromodomain inhibition does not alter IκBα degradation or expression (**Figures 2A, B**) consistent with previous findings that BET bromodomain inhibition does not blunt transcription of all NF-κB targets (43, 44). We also assessed the effects of BET bromodomain inhibition on cyclooxygenase 2 (COX2), a second inflammatory gene induced by IL-1β in an NF-κB-dependent manner (65). IL-1β stimulated transcription of the gene encoding COX2, *PTGS2*, was blunted to control levels by (+)-JQ1 and I-BET151 (**Figure 2C**). This indicates that the requirement of BET bromodomains in NF-κB-driven transcription varies by gene target. Using cell fractionation and immunocytochemistry, (+)-JQ1 did not abolish IL-1β stimulated nuclear translocation of p65 in INS 832/13 cells (**Figures 2D, E**) and quantified by densitometry (**Figure S1A**). These findings suggest that the inhibitory effects of BET bromodomain inhibition on inflammatory gene expression in β-cells do not occur at the level of global NF-κB activation but are focused on transcriptional activation of specific target genes such as *NOS2* and *PTGS2.*

### BET bromodomain inhibition disrupts a p65-BRD4 protein-protein interaction

**Figure 3:**
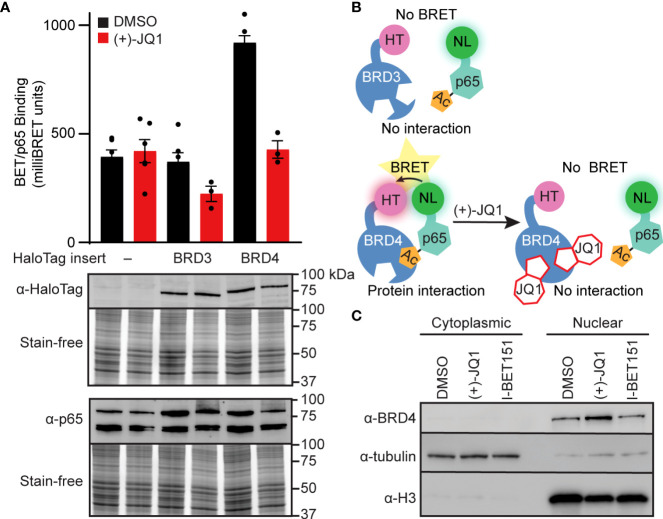
BET bromodomain inhibitors disrupt BRD4-p65 interaction. **(A)** NanoBRET assays completed in INS 832/13 cells transfected with Halo-tagged BET tandem bromodomains and nanoluciferase-tagged p65 exposed to 1 μM (+)-JQ1 or DMSO before BRET intensity reading. Expression blots show relative amounts of transfected proteins. **(B)** Schematic interpretation of NanoBRET results. **(C)** Immunoblot with an antibody directed against BRD4 in INS 832/13 cell fractions after 4-hour exposure to 0.5 μM (+)-JQ1, 1 μM I-BET151, or DMSO. Error bars for nanoBRET assay represent the standard error of the mean (SEM) from 3-6 individually plated wells per treatment condition.

BET bromodomain inhibition may disrupt an interaction between a BET protein and p65, resulting in reduced NF-κB-dependent gene expression. Since BRD4 bromodomains were previously shown to bind to acetylated Lys-310 of p65 in HEK293T cells (43) and BET bromodomain inhibitors disrupt the p65-BRD4 interaction in cancer cells (43, 44), we used nanoluciferase bioluminescence resonance energy transfer (NanoBRET) to quantify the interactions of p65 and BRD4 in INS 832/13 cells. We co-expressed Halo-tagged BET proteins containing only the tandem bromodomains and nanoluciferase-tagged full-length p65 in INS 832/13 cells before exposure to the pan-BET bromodomain inhibitor (+)-JQ1 or DMSO control. We identified that p65 interacts with the tandem bromodomains of BRD4 but not our HaloTag negative control, which lacks bromodomains and therefore is not capable of binding acetyl-lysine residues, or the tandem bromodomains of another BET family member, BRD3. Moreover, the interaction of the BRD4 tandem bromodomains with p65 was disrupted by adding (+)-JQ1 (**Figures 3A, B**). Using subcellular fractionation and immunoblot analysis, we found that native full-length BRD4 is localized exclusively to the nucleus in β-cells. This nuclear location was unaffected by pan-BET bromodomain inhibitor treatment (**Figure 3C**). This further suggests that the ability of BET inhibitors to disrupt NF-κB signaling is enacted at the point of transcriptional activation in the nucleus, rather than upstream signaling cascade components in the cytoplasm. These findings are consistent with BRD4 co-activating transcription of the NF-κB target gene *NOS2* through a bromodomain-dependent interaction with p65 in the nucleus of β-cells.

### BET bromodomain inhibitor-driven increase in SIRT1 protein levels is not required for modulation of IL-1β-induced *NOS2* transcription

**Figure 4:**
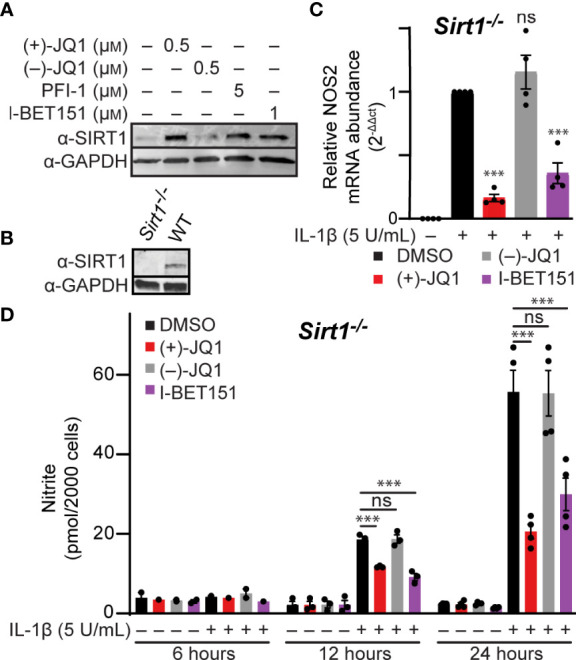
Pan-BET bromodomain inhibitors reduce IL-1β-induced transcription of *NOS2* and NO production independent of SIRT1. **(A)** Immunoblot using an antibody directed towards the SIRT1 protein in INS 832/13 cells treated with indicated pan-BET bromodomain inhibitor or DMSO for 24 hours. **(B)** Immunoblot analysis using antibody directed against SIRT1 protein or GAPDH loading control in WT and *Sirt1* ^-/-^ INS 832/13 cell lines. **(C)** RT-qPCR analysis of *NOS2* transcript levels in *Sirt1* ^-/-^ INS 832/13 cell line treated with 0.5 μM (+)-JQ1, 0.5 μM (–)-JQ1, 1 μM I-BET151, or DMSO and 5 U/mL IL-1β for 12 hours. **(D)** Griess assay for nitrite accumulation in *Sirt1* ^-/-^ INS 832/13 cells treated with 0.5 μM (+)-JQ1, 0.5 µM (–)-JQ1, 1 μM I-BET151, or DMSO, and 5 U/mL IL-1β for 6, 12 or 24 hours. Immunoblots are representative of two independent biological replicates. All error bars for RT-qPCR and Griess assays represent the SEM from 3-5 independent biological replicates, and significance was determined by ANOVA followed by Dunnett’s multiple comparisons tests, *< 0.05, **< 0.01, ***< 0.005, and ns > 0.05.

The pan-BET bromodomain inhibitor (+)-JQ1 was previously shown to elevate SIRT1 protein levels in mouse and human-derived cell lines (66, 67); however, more recent studies indicate that the increase in SIRT1 by pan-BET bromodomain inhibitors is cell type- and inhibitor-dependent (68). SIRT1 can suppress inflammation by deacetylating p65, thereby inhibiting NF-κB activity (69). This has also been shown in β-cells, where SIRT1 overexpression reduces cytokine-driven toxicity by inhibiting NF-κB signaling (70). Therefore, we determined if SIRT1 protein levels increase in BET bromodomain inhibitor-treated β-cells, as this is a potential mechanism through which pan-BET bromodomain inhibitors decrease inflammatory gene transcription. INS 832/13 cells exposed to the pan-BET bromodomain inhibitors (+)-JQ1, I-BET151, and PFI-1 (71) exhibited a marked increase in SIRT1 protein levels following a 24-hour incubation (**Figure 4A** and quantified in **Figure S1B**). To determine if increased SIRT1 levels contribute to BET bromodomain inhibitor-mediated reduction in NF-κB-driven iNOS expression and NO accumulation, we generated a SIRT1 knockout INS 832/13 cell line using CRISPR-Cas9 technology (**Figure 4B**). In SIRT1^-/-^ INS 832/13 cells, (+)-JQ1 and I-BET151 attenuated IL-1β-stimulated *NOS2* mRNA accumulation and nitrite formation (**Figures 4C, D**), similar to wild-type INS 832/13 cells. These data indicate that although pan-BET bromodomain inhibitors increase SIRT1 protein levels in β-cells, SIRT1 does not impact the ability of pan-BET bromodomain inhibitors to reduce NF-κB-driven transcription of *NOS2* in response to the pro-inflammatory mediator IL-1β.

### Inhibition of the first BET bromodomains is sufficient to reduce IL-1β-driven *NOS2* transcription

**Figure 5:**
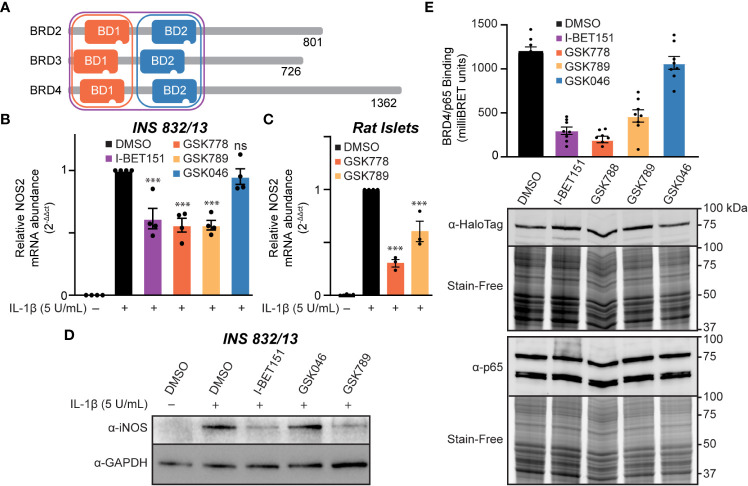
Inhibition of the first BET bromodomains is sufficient to reduce IL-1β-driven *NOS2* transcription. **(A)** Schematic representation of BET bromodomain proteins highlighting the bromodomains inhibited by each inhibitor class. Modified from Gilan et al. (56). **(B)** RT-qPCR analysis of *NOS2* transcript levels in INS 832/13 cells treated for 1 hour with 1 μM indicated BET bromodomain inhibitor followed by IL-1β (5 U/mL) for 3 hours. **(C)** RT-qPCR analysis of *NOS2* transcript levels in rat islets treated for 3 hours with 10 μM indicated BET bromodomain inhibitor followed by IL-1β (5 U/mL) for 3 hours. **(D)** Immunoblot using an antibody directed against iNOS protein in INS 832/13 cells pre-treated with 1 μM indicated BET bromodomain inhibitor followed by IL-1β (5 U/mL) for 20 hours. **(E)** NanoBRET assays completed in INS 832/13 cells transfected with Halo-tagged BRD4 tandem bromodomains and nanoluciferase-tagged p65 exposed to 2 μM indicated BET bromodomain inhibitor followed by BRET intensity reading. Immunoblot for iNOS is representative of two independent biological replicates. For RT-qPCR, error bars represent the standard error of the mean (SEM) from 3-4 independent biological replicates, and significance was determined by ANOVA followed by Dunnett’s multiple comparisons tests, *< 0.05, **< 0.01, ***< 0.005, and ns > 0.05.

We identified that BET family member BRD4 can bind p65 in β-cells *via* its tandem bromodomains (**Figures 3A, B**). Targeted inhibition of individual BET proteins is challenging due to the high degree of structural homology across the BET family (72). A more successful approach has been developing inhibitors selective for either the first or second bromodomain (BD1 or BD2) of all four BET family members (**Figure 5A**) (35, 55, 56, 73). To determine if selective inhibition of either BD1 or BD2 or both is required to decrease IL-1β-induced *NOS2* expression, we examined two BD1-selective inhibitors, GSK778 and GSK789, and the BD2-selective inhibitor, GSK046 (55, 56). INS 832/13 cells were treated with bromodomain selective inhibitors for one hour, followed by a three-hour exposure to IL-1β. The BD1-selective inhibitors GSK778 and GSK789 decreased IL-1β-induced *NOS2* mRNA accumulation by ~50%, similar to pan-BET bromodomain inhibitors, while the BD2-selective inhibitor GSK046 did not affect *NOS2* transcription at concentrations up to 15 μM (**Figures 5B**, **S2**). To confirm that the BD2-selective inhibitor, GSK046, was indeed active in INS 832/13 cells, we validated transcriptional attenuation (TRIM80 and GAS7) and enhancement (MAN1C1) of several BD2-sensitive gene targets by RT-qPCR at 1 µM GSK046 (**Figure S3**). Additionally, we confirmed that BD1-selective inhibition by GSK778 or GSK789 is sufficient to attenuate NOS2 transcription in primary rat islets (**Figure 5C**). Consistent with decreases in mRNA accumulation, pan-BET bromodomain inhibition by I-BET151 and BD1 inhibition by GSK789, but not BD2 inhibition by GSK046, attenuated IL-1β stimulated iNOS expression in INS 832/13 cells as assessed by immunoblot analysis following a 20-hour incubation (**Figure 5D** and quantified in **Figure S1C**). These data support the BD1 bromodomains as the BET bromodomains that need to be inhibited to decrease IL-1β stimulated *NOS2* expression. To further probe this hypothesis, NanoBRET was used to explore whether BD1 of BRD4 is the bromodomain that interacts with p65. Halo-tagged BRD4 tandem bromodomains and nanoluciferase-tagged p65 were co-transfected into INS 832/13 cells and treated with BET bromodomain inhibitors. The pan-BET bromodomain inhibitor I-BET151 and BD1-selective inhibitors GSK778 and GSK789 reduced the interaction between BRD4 and p65 by >50%, whereas BD2-selective inhibition by GSK046 did not affect the p65-BRD4 bromodomain interaction (**Figure 5E**). These data support that BD1 of BRD4 interacts with p65 to co-activate *NOS2* transcription and suggest that inhibition of only BRD4-BD1 may be sufficient to elicit the anti-inflammatory effects of pan-BET bromodomain inhibitors.

### BET bromodomain inhibition mitigates IL-1β-driven mitochondrial deficits and reduced cell viability in β-cells

**Figure 6:**
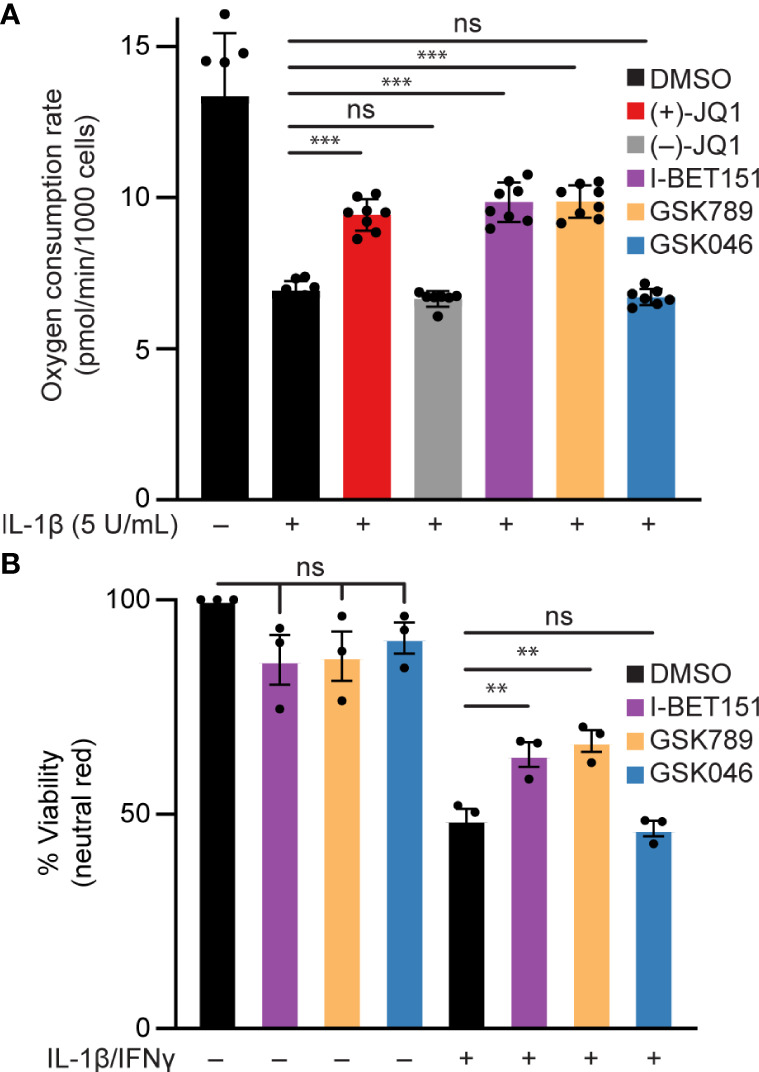
BET bromodomain inhibition prevents IL-1β-induced loss of function in β-cells. **(A)** Basal oxidative consumption rate (OCR) was measured in INS 832/13 cells following 1-hour pretreatment with 0.5 μM (+)-JQ1, 0.5 μM (–)-JQ1, 1 μM I-BET151, 1 μM GSK789, 1 μM GSK046, or DMSO followed by 24-hour treatment with 5 U/mL IL-1β. The mean OCR of 5-6 replicates was plotted with error bars representing SD. **(B)** Neutral red analysis for cell viability in INS 832/13 cells treated for 1 hour with 1 μM indicated BET bromodomain inhibitor followed by IL-1β (5 U/mL) and IFN-γ (150 U/mL) for 24 hours. The mean cell viability was plotted for three independent biological replicates with error bars representing SEM. Significance was determined by ANOVA followed by Dunnett’s multiple comparisons test, *< 0.05, **< 0.01, ***< 0.005, and ns > 0.05.

Elevated or prolonged exposure to IL-1β can lead to decreased β-cell function and increased β-cell death [reviewed in (74)]. Specifically, NO produced following iNOS induction mediates the inhibitory actions of IL-1β on mitochondrial oxidative metabolism resulting in reduced oxygen consumption rate (OCR) (14) and β-cell death (18). Since BET bromodomain inhibitors attenuated the IL-1β-stimulated iNOS expression and NO production, we examined whether this inhibition of NO production is sufficient to prevent the inhibitory actions on mitochondrial oxidative metabolism and cell death in β-cells. IL-1β decreased the OCR as determined by extracellular flux analysis (**Figure 6A**) and β-cell viability as determined by the neutral red assay (**Figure 6B**), and these responses were attenuated by pretreatment with pan-BET bromodomain inhibitors (+)-JQ1 or I-BET151. These actions are due to BD1 targeting, as the BD1-selective inhibitor GSK789 also attenuated the inhibitory activities of IL-1β on the OCR and β-cell viability to levels similar to those observed for pan-BET bromodomain inhibitors (**Figure 6**). As anticipated, BD2-selective inhibition by GSK046 did not modify the inhibitory actions of IL-1β on the OCR and β-cell viability (**Figure 6**). Taken together, BD1-selective inhibition is sufficient to partially protect against IL-1β-driven mitochondrial defects and β-cell death linked to high NO levels.

## Discussion

This study describes a mechanism by which inhibition of a specific BET bromodomain reduces *NOS2* transcription and β-cell dysfunction in response to IL-1β, a cytokine known to inhibit β-cell function. Previous studies demonstrated that pan-BET bromodomain inhibitors protect against insulitis in NOD mice (37, 38). However, the direct actions of these inhibitors on β-cells had yet to be examined in detail. Further, the role of individual bromodomains and specific isoforms of BET proteins in regulating inflammatory gene expression in β-cells had yet to be determined.

Cytokine-induced β-cell damage is believed to contribute to the loss of functional β-cell mass during the development of autoimmune diabetes. Cytokines damage β-cells by stimulating the expression of iNOS and the production of micromolar NO levels [reviewed in (75)]. Because iNOS expression is an NF-κB-dependent process and BET-containing proteins interact with the p65 subunit of NF-κB, we hypothesized that BET bromodomain inhibition might prevent β-cell inflammatory responses and dysfunction by attenuating IL-1β-induced transcriptional activation of *NOS2.* Using INS 832/13 cells and isolated rat islets exposed to IL-1β, we show that pan-BET bromodomain inhibitors attenuate IL-1β-induced iNOS expression and NO production by decreasing NF-κB activation (**Figure 1**). Administration of the pan-BET bromodomain inhibitor I-BET151 to NOD mice attenuates the transcription of select NF-κB target genes in β-cells and islet-resident macrophages (37). Using a luciferase-based NF-κB reporter assay, we demonstrate that pan-BET bromodomain inhibitors decrease NF-κB reporter activity (**Figure 1H**). BET bromodomain inhibitors do not halt cytoplasmic NF-κB signaling cascades, as IL-1β-induced IκBα degradation and p65 translocation to the nucleus are unchanged by BET bromodomain inhibition (**Figures 2A, D, E**). Instead, we show that BET bromodomain inhibitors disrupt a BRD4 tandem bromodomain-p65 interaction, likely in the nucleus at the point of transcriptional activation (**Figures 2**, **3**). The interaction between p65 and BRD4 has previously been observed in HEK293T and A549 cancer lines (43, 44) but not in β-cells. One potential mechanism by which BRD4 is involved in the transcriptional activation of *NOS2* is to assist in overcoming paused RNA polymerase II (pol II) (76, 77), a transcriptional control feature often associated with inducible genes. In support of this, BRD4 contains a unique *C*-terminal motif absent in the other BET proteins and that can recruit P-TEFb, leading to phosphorylation of RNA pol II and rapid activation of transcriptional elongation (43, 78–81). To identify which of the BRD4 tandem bromodomains interacts with p65, we paired bromodomain-specific inhibitors with NanoBRET binding assays. We determined that BRD4 interacts with p65 exclusively *via* the first (BD1) bromodomain (**Figure 5E**).

We also addressed an alternative mechanism by which BET inhibitors could reduce *NOS2* transcription. Previous studies identified BET bromodomain inhibitors can increase SIRT1 protein levels in non-β-cells (66, 68), potentially an effective inhibitor of inflammatory signaling and modulator of NF-κB transcriptional activation through SIRT1-mediated deacetylation of p65 (66, 69, 70). We found that although BET bromodomain inhibitors increase SIRT1 expression levels in β-cells (**Figure 4A**), the reduced IL-1β-driven transcription of *NOS2* was independent of SIRT1 expression (**Figure 4**). Nevertheless, additional gene targets may be induced by the increase in SIRT1 protein levels that would be beneficial in the context of T1D. The SIRT1 activator resveratrol has been demonstrated to attenuate diabetes development in NOD mice (82), and familial mutations in SIRT1 are associated with an increased risk for T1D development in humans (83). One beneficial role for SIRT1 in the context of diabetes, which requires further investigation, is the potential enhancement of insulin secretion (84, 85).

An additional mechanism by which BET inhibitors may provide beneficial effects in the context of T1D is *via* attenuation of the senescence-associated secretory phenotype (SASP). Indeed, the pan-BET inhibitor I-BET762 attenuated SASP in islet cells and prevented diabetes in NOD mice (38). As such, follow-up studies assessing the role of BRD4, particularly the consequences of inhibiting BD1, in SASP in β-cell should be evaluated.

The requirement of BET proteins is specific to only some promoter regions. Even within NF-κB target genes, only select NF-κB targets are downregulated by BET bromodomain inhibition while others are unaffected (43, 44), as shown here by reduced transcription of *NOS2* and *PTGS2* but not *NFKBIA* (**Figures 1**, **2**). Additionally, the ability of BET bromodomain inhibitors to impact only a subset of NF-κB targets has been shown in NOD mice, where the NF-κB inhibitor BAY 11-7082 reduces transcription of more NF-κB gene targets than I-BET151 (37). This disparate effect is likely because BAY 11-7082 blocks IκBα phosphorylation (86), thereby inhibiting an earlier step in NF-κB signaling. In contrast, BET bromodomain inhibition affects the transcriptional activation of NF-κB targets with unknown specific promoter requirements. In theory, blunting some but not all NF-κB gene targets is beneficial; global, untargeted inhibition of the entire NF-κB signaling pathway for any significant duration would have toxic, dose-limiting effects (87). Equally intriguing are the NF-κB-independent genes whose gene expression is regulated by BET bromodomain inhibition. Other inflammatory status and β-cell identity markers may be modulated by BET bromodomain inhibition and may provide additional benefits in specific inflammatory contexts (37).

**Figure 7:**
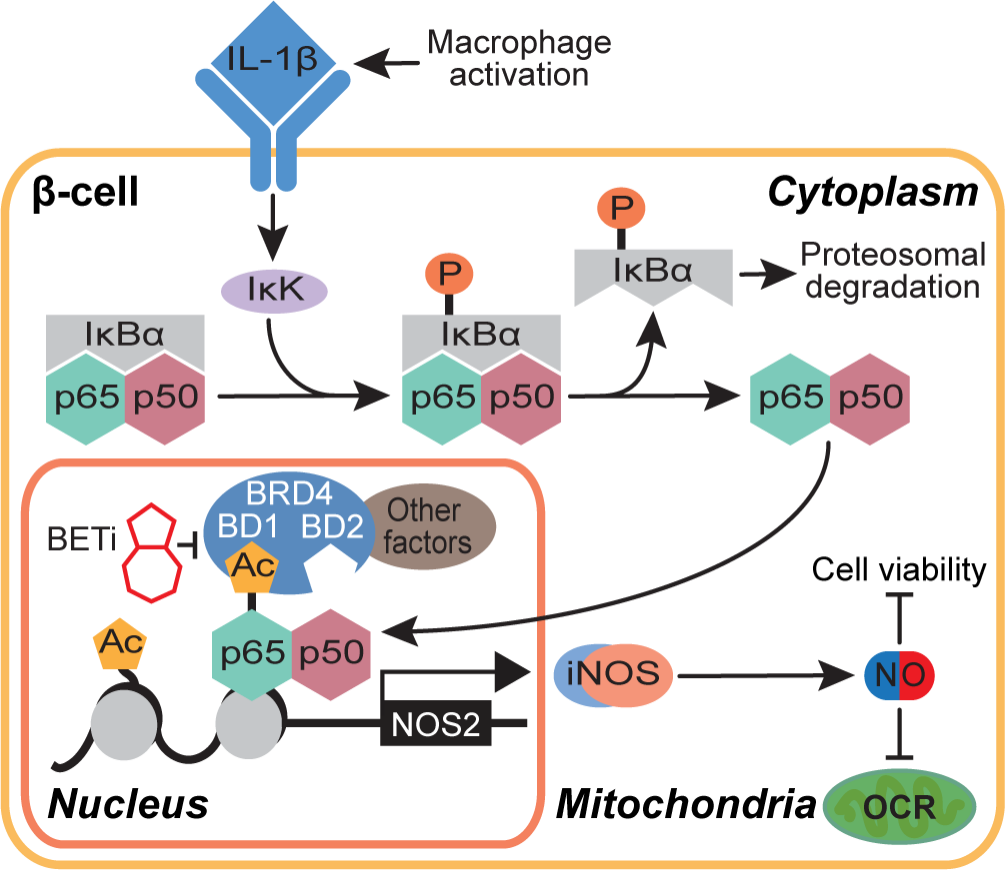
Schematic representation of the BET bromodomain inhibitor-driven decrease in NOS2 transcriptional activation mechanism.

In summary, this study proposes a role for BRD4 in NF-κB-driven gene transcription in β-cells. We identify the first bromodomain of BRD4 as a p65 binding partner and BRD4 as a potential transcriptional co-activator of p65. These findings also provide mechanistic insights into how BET bromodomain inhibition produces a protective role in β-cells exposed to an inflammatory insult. Selective inhibition of only the first bromodomain of BRD4 disrupts the interaction between p65 and BRD4 (**Figure 5E**) and reduces IL-1β-driven transcription of *NOS2* (**Figures 1**, **5**). This attenuates the NO-mediated loss of OCR and decrease in β-cell viability (**Figure 6**), thus limiting β-cell dysfunction (**Figure 7**). By identifying a single bromodomain involved in a possible co-activation of select p65 target genes, we lay the groundwork for ‘next-generation’ compounds selective for a single BET bromodomain. Several industrial groups are currently developing next-generation BET inhibitors with selectivity for subsets of BET bromodomains (55, 56, 73) with the goal of increased safety profiles in clinical trials. Although BD2-selective inhibitors are being evaluated in clinical trials, there are currently no clinical trials accessing the effectiveness of BD1-selective compounds [reviewed in (88)]. However, this study suggests that BRD4-BD1 selective inhibitors are particularly desired in this context to prevent T1D. Importantly, BET bromodomain inhibitors with selectivity for BRD4-BD1 were recently reported (89), which will be interesting to assay for activity in diabetes contexts. Such compounds may be highly efficacious without dose-limiting adverse effects witnessed in clinical trials where pan-BET bromodomain inhibitors have been used [reviewed in (39)], especially in diseases like T1D where patient populations are primarily pediatric.

## Data availability statement

The original contributions presented in the study are included in the article/**Supplementary Material**. Further inquiries can be directed to the corresponding author.

## Ethics statement

The animal study was reviewed and approved by Institutional Animal Care and Use Committee at the Medical College of Wisconsin.

## Author contributions

JN designed experiments, performed experiments, analyzed data, and wrote the manuscript. BS designed experiments and contributed to data analysis/interpretation. SW-S performed NanoBRET and OCR experiments, performed immunoblots, assisted with rat islet isolation, and analyzed data. AN generated and characterized the *Sirt1* ^-/-^ INS 832/13 cell line. RJL performed immunoblots. AG completed immunofluorescence micrographs. RP and IR provided bromodomain-selective inhibitors and provided helpful discussion and guidance. JC provided critical discussion and guidance. All authors edited the manuscript and approved the final submission.

## Funding

This work was supported by NIH grants R01 DK119359 (BS), T32 HL134643 (JN), R01 DK052194 (JC), and R01 AI044458 (JC), and the Cardiovascular Center’s A.O. Smith Fellowship Scholars Program (JN).

## Acknowledgments

We thank Drs. Moua Yang and Michael Olp for their assistance in initiating this project. We thank Monika Zielonka of the MCW Cancer Center Redox and Bioenergetics Shared Resource Center for technical assistance in conducting Seahorse assays.

## Conflict of interest

RP and IR: GlaxoSmithKline is interested in the potential therapeutic applications of BET bromodomain inhibitors.

The remaining authors declare that the research was conducted in the absence of any commercial or financial relationships that could be construed as a potential conflict of interest.

## Publisher’s note

All claims expressed in this article are solely those of the authors and do not necessarily represent those of their affiliated organizations, or those of the publisher, the editors and the reviewers. Any product that may be evaluated in this article, or claim that may be made by its manufacturer, is not guaranteed or endorsed by the publisher.
