# Supplementary material for: N-terminal BET bromodomain inhibitors disrupt a BRD4-p65 interaction and reduce inducible nitric oxide synthase transcription in pancreatic β-cells
Source: Front Endocrinol (Lausanne). 2022 Sep 13;13:923925. doi: 10.3389/fendo.2022.923925 (PMC9513428; doi:10.3389/fendo.2022.923925)
Supplement: Supplementary file 1 [file DataSheet_1.pdf]

Supplementary Material

Supplementary Table 1. Primer sequences used for RT-qPCR experiments.

Gene target	Forward primer (5′-3′)	Reverse primer (5′-3′)
NOS2	CGAGACTTCTGTGACACACAGC	CATCTCCTGGTGGAACACAGGG
GAPDH	GACATCAAGAAGGTGGTGAAGC	TCCAGGGTTTCTTACTCCTTGG
NFKBIA	GAGGATTACGAGCAGATGGTG	ACCTGACCAATCACTTCCATG
PTGS2	TTTGTTGAGTCATTCACCAGACAGAT	CAGTATTGAGGAGAACAGATGGGATT

Supplementary Table 2. Antibodies used for immunoblotting and immunochemistry experiments.

**Target protein**	**Supplier**	**Catalog number**	**RRID number**	**Lot/batch**	**Application and dilution**
iNOS	Cayman Chemical	160862	AB_10079372	0560942-1	Immunoblot: 1:500
NF-κB p65	Cell Signaling Technology	8242	AB_10859369	8 and 16	Immunoblot: 1:1000<br>Immunocytochemistry: 1:400
IκBα	Cell Signaling Technology	9242	AB_331623	11	Immunoblot: 1:2000
BRD4	Abcam	ab128874	AB_11145462	GR275920-34	Immunoblot: 1:1000
SIRT1	Abcam	ab110304	AB_10864359	GR3200692-2	Immunoblot: 1:1000
α-tubulin	Sigma	T9026	AB_477593	029M-4880V	Immunoblot: 1:5000
GAPDH	ThermoFisher Scientific	AM4300	AB_2536381	00777308	Immunoblot: 1:5000
H3	Abcam	ab1791	AB_302613	GR3297884-1	Immunoblot 1:2000

Supplementary Table 3. BET bromodomain inhibitors used in this study.

Compound name	Structure	Supplier
(+)-JQ1	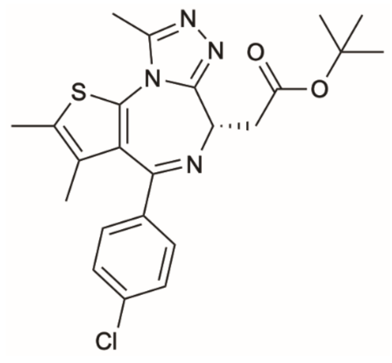	eNovation
(–)-JQ1	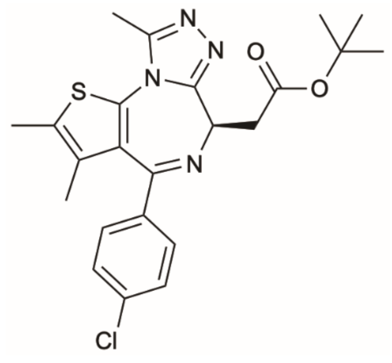	eNovation
I-BET151	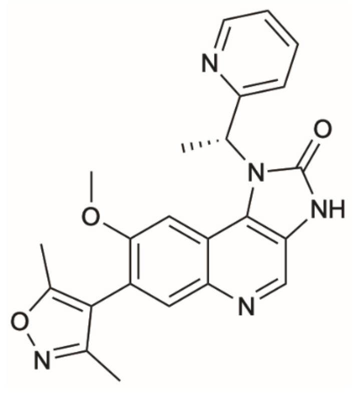	A gift from GlaxoSmithKline
PFI-1	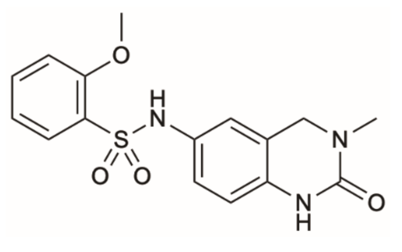	ApexBio

GSK-778	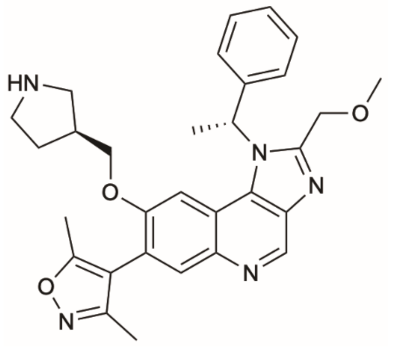	A gift from GlaxoSmithKline
GSK-789	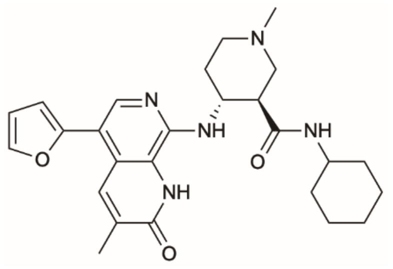	A gift from GlaxoSmithKline
GSK-046	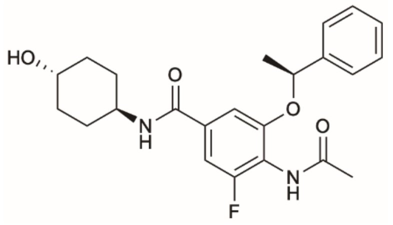	A gift from GlaxoSmithKline

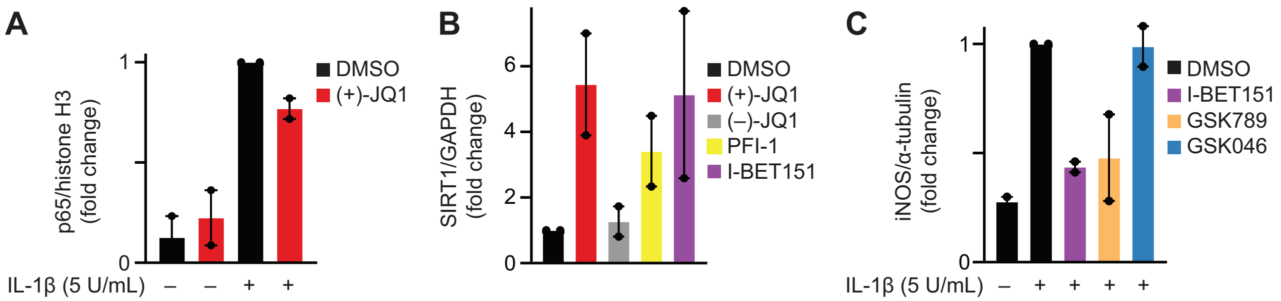

Supplementary Figure S1. Quantification of select immunoblots. (A) Quantification of p65/histone H3 ratio in INS 832/13 nuclear fractions correlating to the representative immunoblot displayed in Figure 2D. **(B)** Quantification of SIRT1/GAPDH ratio in INS 832/13 whole cell lysates correlating to the representative immunoblot displayed in Figure 4A. **(C)** Quantification of iNOS/α-tubulin ratio in INS 832/13 whole cell lysates correlating to the representative immunoblot displayed in Figure 5D. All immunoblot quantifications depict two independent biological replicates and adjusted band volumes of the appropriate bands were acquired via Image Lab software (Bio-Rad).

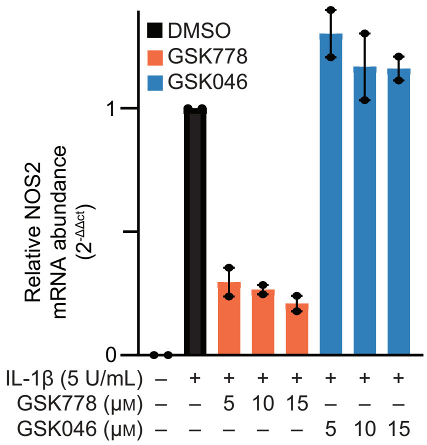

Supplementary Figure S2. RT-qPCR analysis of *NOS2* in INS 832/13 cells treated for 1 hour with the indicated BET bromodomain inhibitor and concentration followed by addition of 5 U/mL IL-1β for 3 hours.

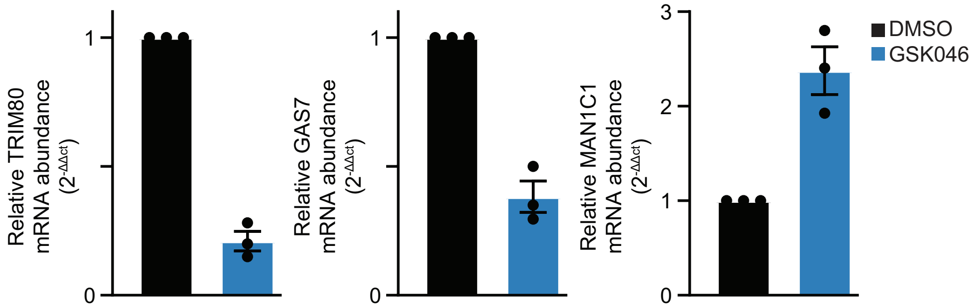

Supplementary Figure S3. RT-qPCR analysis of select genes following 24-hour treatment with 1 μM GSK046 or DMSO control.
